# Detection of drug effects on gastric emptying and contractility using a wireless motility capsule

**DOI:** 10.1186/1471-230X-14-2

**Published:** 2014-01-02

**Authors:** Inna Rozov-Ung, Amjad Mreyoud, John Moore, Gregory E Wilding, Elias Khawam, Jeffrey M Lackner, John R Semler, Michael D Sitrin

**Affiliations:** 1 Division of Gastroenterology, Hepatology, and Nutrition, University at Buffalo, State University of New York, 462 Grider, Rm 132-7, Buffalo, NY 14215, USA; 2 Department of Biostatistics, University at Buffalo, Buffalo, USA; 3 SmartPill Corporation, Buffalo, NY, USA

**Keywords:** Wireless motility capsule, Erythromycin, Morphine, Gastric motility

## Abstract

**Background:**

A wireless motility capsule is a new method for ambulatory assessment of transit times and motility throughout the gastrointestinal tract. The objective of this study was to evaluate the ability of a wireless motility capsule to detect drug effects on gastric emptying time (GET) and gastric contractility.

**Methods:**

15 healthy adults were administered in random order saline, erythromycin IV 150 mg, or morphine IV 0.05 mg/kg BW. Subjects ate a standard meal after each infusion, and subsequently ingested the motility capsule. Data were recorded for 8 hours, and the results were analyzed using the manufacturer’s software.

**Results:**

GET was significantly faster after erythromycin than either saline or morphine. Morphine tended to delay emptying of the capsule compared to saline. There was a trend toward a greater frequency of gastric contractions with erythromycin and a reduced frequency of gastric contractions with morphine that did not reach statistical significance.

**Conclusions:**

A wireless motility capsule successfully detected acceleration of gastric emptying induced by erythromycin, and retardation of gastric motility caused by morphine. These results indicate that a wireless motility capsule is a promising technique to assess pharmacologic effects on gastric transit and contractility and aid in development of drugs for gastric motor disorders.

## Background

Disorders of gastric motility are commonly encountered in clinical practice. Gastroparesis results from a variety of neuropathic and myopathic disorders, most commonly diabetes mellitus, post-gastric or esophageal surgery, or as an idiopathic abnormality often following a viral infection [1]. Delayed gastric emptying also occurs in some patients with gastroesophageal reflux disease and functional dyspepsia [2]. Accelerated gastric emptying may result from gastric surgery, causing the dumping syndrome, and also is observed early in the course of diabetes [3,4]. Both delayed and accelerated gastric motility can significantly interfere with a patient’s quality of life. Upper gastrointestinal symptoms such as nausea, vomiting, and abdominal pain are frequent side effects of many medications, and may be due in part to drug effects on upper gastrointestinal tract transit and contractility. Evaluation of gastric motility is usually performed by gastric scintigraphy or ^13^C-labeled acetate and octanoic acid breath tests [5]. Gastric scintigraphy involves a 12-hour fast and subsequent ingestion of a standardized meal labeled with a radioactive isotope. Imaging of the radio-labeled meal tracks its course throughout the upper gastrointestinal tract. Studies have shown that the test should be conducted for 4 hours to optimize the ability to distinguish normal from delayed gastric emptying [6]. The exposure of volunteer subjects to the radioactive meal and the need for access to nuclear medicine facilities for a prolonged time can be problematic for drug development research. The accuracy of the ^13^C -labeled acetate and octanoic acid breath tests is comparable to scintigraphy, however the use of these tests is also restricted by limited availability and expense of mass spectrometry resources. A method to safely assess upper gastrointestinal tract motility in ambulatory subjects would be of significant benefit in the development of new treatments for gastric motor disorders and in the assessment of upper gastrointestinal side effects of drugs in general.

In this study, we have tested the hypothesis that an ambulant wireless motility capsule (SmartPill^R^, SmartPill^R^ Corporation, Buffalo, NY) can detect changes in gastric emptying and motor activity induced by moderate doses of erythromycin and morphine.

## Methods

### Subjects

The subjects were 15 healthy adults, 12 males and 3 females ages 19 to 65. All subjects were evaluated with a medical history, complete physical examination, electrocardiogram, complete blood count, comprehensive metabolic panel, and thyroid stimulating hormone level prior to receiving any infusions. Pregnancy tests were performed in females of child bearing potential prior to each infusion. No smoking or alcohol use was allowed for at least 24 hours prior to each visit. Exclusion criteria included chronic cardiovascular disease, chronic pulmonary disease, morbid obesity, sleep apnea, chronic gastrointestinal or liver disease, history of gastrointestinal surgeries, diabetes, hyperthyroidism, hypothyroidism, renal or urinary tract disease, and pregnancy or lactation. Patients with an allergy to any of the study medications or components of the test meal and those taking medications which may affect bowel motility (narcotics, anticholinergics, calcium channel blockers) or gastric pH (proton pump inhibitors, H_2_ receptor blockers) were also excluded.

### Study protocol

The subjects were evaluated at the study center on three different occasions separated by at least one week. In random order, each subject was administered an intravenous infusion of either 100 cc of saline (placebo), erythromycin 150 mg in saline, or morphine 0.05 mg/kg of body weight in saline. Each infusion was given over a 20-minute period. The doses of medication were selected to produce a moderate effect on gastric emptying based on prior studies utilizing other methods for studying gastrointestinal transit [7,8]. Subjects were not aware of the infusion they received on a given test day.

At the completion of the infusion, the subject ingested a standardized meal consisting of a nutrient bar (SmartBar^R^) that is composed of 66% carbohydrates, 17% protein, 2% fat and 3% fiber. Subsequently, the subject ingested the activated SmartPill^R^, which records pressure and pH data and transmits it to a SmartPill^R^ Data Receiver worn by the patient. After capsule ingestion, the subjects were observed in the study center for 8 hours, throughout which all data were recorded. Six hours after capsule ingestion, each subject was allowed to drink 1 bottle of Ensure^R^ nutritional drink. Subjects either retrieved and returned the SmartPill^R^ capsule or had an abdominal x-ray in 1 week to document capsule passage from the body. The protocol was approved by the University at Buffalo and VA Western New York IRBs. Written informed consent for participation in the study was obtained from the subjects.

### SmartPill^R^ technology

The system includes a wireless motility capsule, data receiver, and data analysis software GIMS Data Viewer v.1.4 and SmartPill MotiliGI, which are provided by the manufacturer. The capsule size is 26 mm × 13 mm, similar to the Given^R^ endoscopy capsule. The SmartPill^R^ contains sensors that measure pH, pressure, and temperature. pH was measured every 5 seconds, and pH changes from 0.5 to 9.0 were detected with a sensitivity of ±0.5 pH units. Pressure measurements from 0–350 mmHg were acquired every 0.5 second, and temperatures from 25-49°C obtained every 20 seconds. At the end of each 8-hour study period, data were downloaded to a laptop computer for analysis.

### Data analysis

Gastric emptying time (GET) was defined as the time from capsule ingestion to an abrupt and sustained rise in pH ≥ 2 units from the lowest postprandial value to ≥ 4, which indicates that the capsule exited the acidic gastric antrum and entered the alkaline duodenum. The frequency, area under the pressure curve (AUC) and motility index of contractions during capsule residence in the stomach were evaluated using both 10 mmHg and 20 mmHg above baseline as the minimal contraction threshold. The motility index was calculated as described by Camilleri et al.: MI = Ln(sum of pressure amplitudes * number of contractions +1) [9]. The analyses of the GETs and motility data were performed by investigators who were not aware of the test infusion conditions.

### Statistical analysis

GETs great than 6 hours, the time at which the nutritional drink was given, were treated as censored in the analysis, and estimates of mean and standard deviation were based on an assumed normal-based model. Two-sided exact sign tests which do not depend on the actual GET values, but rather the relative ordering were used to compare GETs between the three test infusions. In the analysis of average gastric contractions a linear model was utilized. The log transformation was first applied in order to meet statistical assumptions. Once the model was fit, specific linear contrasts based on the estimated model parameters were constructed and used to statistically compare groups in a pairwise fashion. A nominal significance level of 0.05 was used in all testing and all analyses were carried out using SAS version 9.1.3 statistical software (Cary, NC).

## Results

All 15 subjects completed the entire study, and were evaluated under all three experimental conditions. The subjects experienced no adverse events from the study drugs, and were unable to tell whether they were receiving saline, erythromycin, or morphine.

### Gastric emptying time

**Figure 1:**
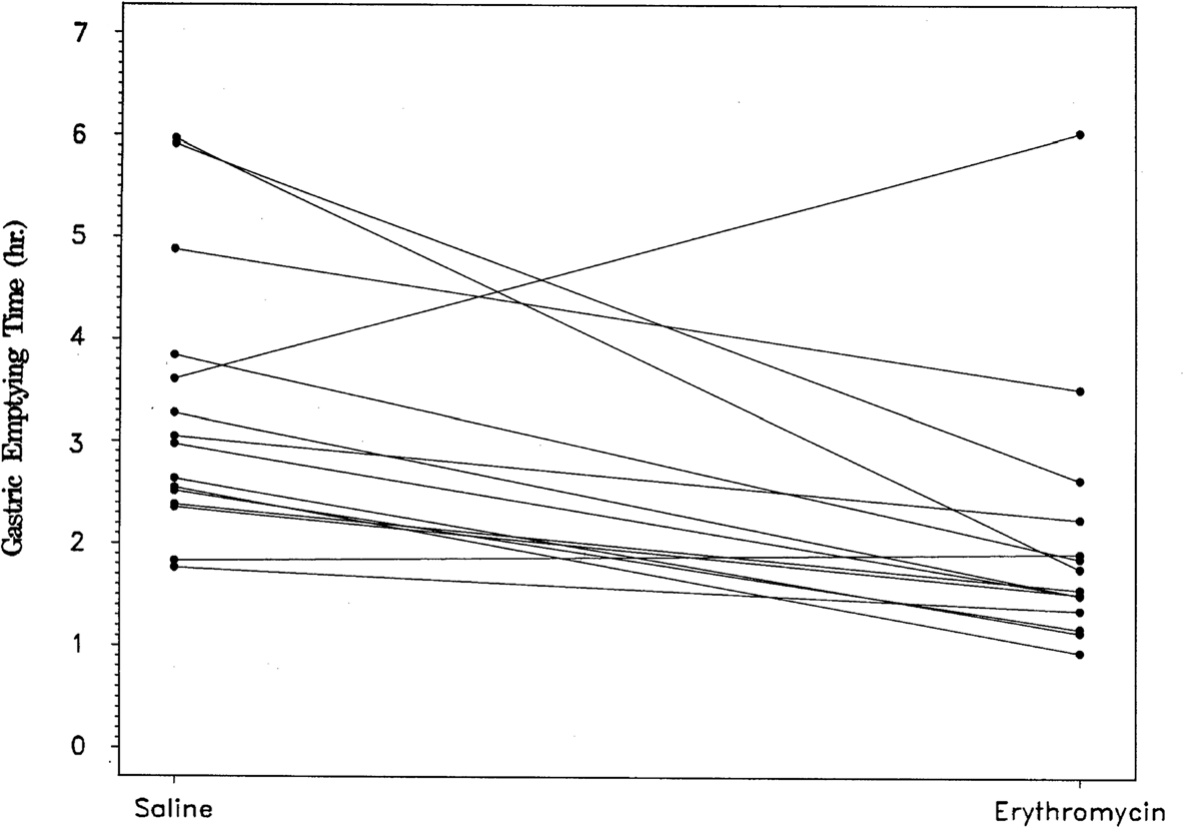
**Individual subject gastric emptying times after administration of saline or erythromycin.** Gastric emptying of the capsule was more rapid after erythromycin than saline in 13 of 15 subjects, p < 0.001.

**Figure 2:**
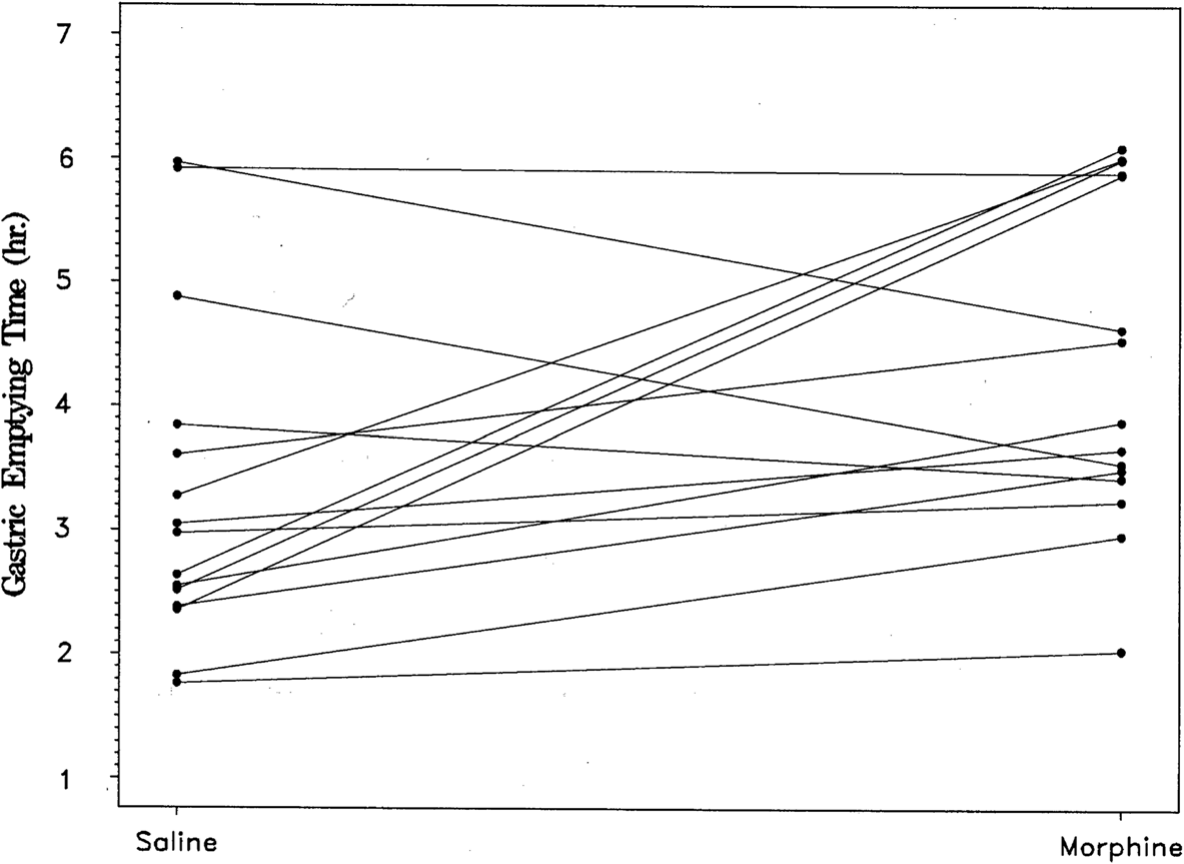
**Individual subject gastric emptying times after administration of saline or morphine.** Gastric emptying of the capsule was slower after morphine than saline in 11 of 15 subjects, p = 0.11.

Table 1 shows the estimated mean GET in hours for each study condition. In 6 of the 45 tests (2 saline, 1 erythromycin, and 3 morphine), the capsule did not empty the stomach within 6 hours, prior to ingestion of the liquid formula meal. Although there were censored observations present, the relative order of GETs for each subject under the three test conditions was still identifiable. Differences in GET between the conditions, therefore, were tested via two-sided exact sign tests. After receiving erythromycin, the subjects had a mean gastric emptying time of 2.07 hours, which was faster than the GET of 3.38 hours after the saline infusion. In 13 of the 15 subjects, GET was faster when the subject received erythromycin compared to the saline placebo (p < 0.001, Figure 1). In contrast, mean GET after morphine administration was longer than after saline, 4.52 hours versus 3.38 hours (Table 1). In 11 of the 15 subjects, GET was slower after the morphine infusion than after saline (p = 0.11, Figure 2). The GET after erythromycin was shorter than after morphine in 14 of 15 subjects (p <0.01), with a mean difference of almost 3 hours between the two medications (Table 1).

**Table 1:** Effects of erythromycin and morphine on mean gastric emptying time

**Drug**	**Mean GET in hours ± SD**
Erythromycin	2.07 (**±** 1.32)
Saline	3.38 (**±** 1.47)
Morphine	4.52 (**±** 1.55)

### Contractility

The frequency of gastric contractions was assessed under the three experimental conditions during the entire time the motility capsule was in the stomach, up to a maximum of 6 hours at which time the liquid nutritional formula was ingested. Data were analyzed using both 10 mmHg and 20 mmHg above baseline as the minimum pressure threshold for a contraction. With the 10 mmHg threshold there was no significant effect of either erythromycin or morphine on gastric contraction frequency (Table 2). Using the 20 mmHg threshold there was a trend for the frequency of gastric contractions to be reduced with morphine compared to either saline or erythromycin that did not reach statistical significance (Table 2). Gastric contraction frequency was lower with morphine than with either saline or erythromycin in 10 of the 15 subjects. Gastric contraction frequency after erythromycin administration was greater than that seen with saline in 10 of the 15 subjects, but because of great variability in the contraction frequency observed with erythromycin there was no statistical difference in the mean values.

**Table 2:** Mean number of contractions per minute during the entire gastric residence of the capsule under different experimental conditions

**Drug**	**Contraction frequency (SD) (minimum contraction threshold = 10 mmHg)**	**Contraction frequency (SD) (minimum contraction threshold = 20 mmHg)**
Erythromycin	1.14 (± 1.02)	0.48 (± 0.58)
Saline	1.10 (± 0.74)	0.37 (± 0.26)
Morphine	0.94 (± 0.48)	0.29 (± 0.16)*

*p = 0.14 morphine vs erythromycin, p = 0.12 saline vs morphine.

Using both a 10 mmHg and 20 mmHg contraction threshold, neither erythromycin nor morphine had a significant effect on area under the pressure curve or motility index.

## Discussion

A wireless motility capsule is being increasingly used for the assessment of transit time and motility throughout the gastrointestinal tract. The capsule contains pH, temperature, and pressure sensors that permit detection of emptying of the capsule from the stomach into the duodenum, transition from the ileum to the right colon [10], and exit from the body, as well as assessment of contractions in different segments of the gastrointestinal tract. Previous studies have shown a good correlation between gastric emptying of a radiolabeled meal and gastric residence time of the capsule [11]. Furthermore, significant differences in gastric emptying time of the capsule were observed between healthy controls and patients with a history of gastroparesis [12]. The wireless motility capsule also was capable of detecting differences in the frequency and motility index of antral and duodenal contractions in patients with severe gastroparesis compared to healthy controls or patients with mild to moderate gastroparesis [13]. A wireless motility capsule can also be used to assess transit time through the whole gut, small intestine, and large intestine, and whole gut and colonic transit times have been shown to correlate well with conventional radioopaque markers [14]. Movement of the capsule through the colon is delayed in patients with slow transit constipation as defined by radioopaque markers [14]. A wireless motility capsule also correlates well with whole gut scintigraphy in the evaluation of whole gut transit time [15].

In the present study, we have tested the hypothesis that a wireless motility capsule can measure drug effects on gastrointestinal motility. In this proof of principle study, we used agents that typically accelerate (erythromycin) or slow (morphine) gastric emptying. We selected doses of these agents that produced modest effects on gastric emptying time that would be most relevant to drugs in clinical practice or pharmaceutical development [7,8]. Administration of 150 mg of erythromycin which acts as a motilin receptor agonist caused a mean decrease of capsule emptying time of about 90 minutes compared to the saline control, and gastric emptying was more rapid after erythromycin than after saline administration in 13 of 15 subjects. In contrast, morphine resulted in delay of gastric emptying time of more than one hour compared to saline. The effect of morphine on gastric emptying time was somewhat variable, with the capsule emptying being delayed by morphine administration in 11 of the 15 test subjects. The variability may be explained, in part, by complex effects of morphine on gastrointestinal motor function, as opiates interfere with both stimulatory and inhibitory neural pathways [16,17]. Although morphine usually delays gastric emptying, previous studies found that narcotics can cause phasic contractions, including some that mimic phase III migrator motor complexes, that could sometimes result in emptying of the capsule from the stomach [16,17].

Cassilly, et al. simultaneously used antroduodenal catheter manometry, gastric emptying scintigraphy, and a wireless motility capsule to study gastric emptying and upper gastrointestinal tract motility in normal volunteers [18]. They found that approximately 2/3 of patients emptied the wireless motility capsule from the stomach with either the first or second phase III migrating motor complex (MMC); 1/3 of patients emptied it with isolated antral contraction*s*. Thus, differences in the type of contraction that propelled the capsule on the three test days also likely contributed to some of the variability in drug effects on capsule emptying observed in this study. In prior studies using the wireless motility capsule employed in this paper, 5–10 percent of healthy subjects with normal emptying of a meal retained the capsule in the stomach for greater than 6 hours [11-13]. This difference between emptying of a meal and a solid inert object is a limitation of the use of wireless capsule manometry.

In evaluating the ability of a wireless motility capsule to detect pharmacologic effects on gastric contractility, we found a numerical trend towards an increased number of contractions after administration of erythromycin and a decreased frequency of gastric contractions with the administration of morphine, particularly using 20 mmHg above baseline as the contraction threshold. These effects, however, were quite variable and not statistically significant. Studies using antroduodenal manometry to assess the effects of erythromycin and morphine on gastric contractions have also demonstrated complex dose-dependent actions [19-21]. Further studies of the role of a wireless motility capsule in assessing the effects of drugs on gastric contraction patterns will need a larger numbers of subjects, more detailed analyses than simple measures of contraction frequency and amplitude, and evaluation of dose-dependent responses.

None of our study patients suffered any adverse effects as a result of this study, including prolonged capsule retention. Data from studies of the device used for video capsule endoscopy that is of similar size have shown that it is a safe and well-tolerated procedure [22].

## Conclusions

The results of this study indicate that a wireless motility capsule may be a very useful tool for assessing actions of drugs on upper gastrointestinal tract motility. Using a modest number of subjects, we demonstrated that the technique can measure drug effects on gastric emptying time. Wireless capsule motility is performed on an ambulatory basis and avoids disadvantages inherent in gastric scintigraphy, such as radiation exposure and a long test period in a nuclear medicine facility which can be problematic for research volunteers. ^13^C-labeled acetate- and -octanoic acid breath tests are limited by the cost and availability of mass spectrometry resources. Evaluation of the effects of pharmacologic agents on gastric emptying using a wireless motility capsule may be an important initial step in the development of new drugs for the treatment of gastric motor disorders before embarking on clinical trials requiring large numbers of subjects.

## Competing interests

Dr. Sitrin and Dr. Lackner serve as speakers, consultants, or advisory board members for the SmartPill Corporation, and have receiving research funding from the Smart Pill Corporation. Dr. Wilding serves as a consultant to the SmartPill Corporation. Dr. Semler is an employee of the SmartPill Corporation and owns stock in the SmartPill Corporation.

## Authors’ contributions

IR performed the research, analyzed the data, and wrote the paper. AM performed the research and analyzed the data. JM performed the research and analyzed the data. GEW analyzed the data and wrote the paper. EK performed the research. JML designed the research study, analyzed the data, and wrote the paper. JRS designed the research study, analyzed the data, and wrote the paper. MDS designed the research study, performed the research, analyzed the date and wrote the paper. All authors read and approved the final manuscript.

## Pre-publication history

The pre-publication history for this paper can be accessed here:

http://www.biomedcentral.com/1471-230X/14/2/prepub
